# Chinese family with diffuse oesophageal leiomyomatosis: a new *COL4A5/COL4A6* deletion and a case of gonosomal mosaicism

**DOI:** 10.1186/s12881-015-0189-7

**Published:** 2015-07-16

**Authors:** Wei Liu, John KL Wong, Qiuming He, Emily HM Wong, Clara SM Tang, Ruizhong Zhang, Man-ting So, Kenneth KY Wong, John Nicholls, Stacey S Cherny, Pak C Sham, Paul K Tam, Maria-Mercè Garcia-Barcelo, Huimin Xia

**Affiliations:** Department of Pediatric Surgery, Guangzhou Women and Children’s Medical Center, Guangzhou, China; Department of Psychiatry, The University of Hongkong, Hongkong, SAR China; Department of Surgery, The University of Hongkong, Hongkong, SAR China; Department of Pathology, The University of Hongkong, Hongkong, SAR China; Center for Genomic Sciences, The University of Hongkong, Hongkong, SAR China; Centre for Reproduction, Development, and Growth of the Li Ka Shing Faculty of Medicine, Hong Kong, SAR China; State Key Laboratory of Brain and Cognitive Sciences, The University of Hong Kong, Hong Kong, SAR China

**Keywords:** Isolated diffuse oesophageal leiomyomatosis, Whole exome sequencing, Copy number variation, *COL4A5*, *COL4A6*, Gonosomal mosaicism

## Abstract

**Background:**

Diffuse oesophageal leiomyomatosis (DOL) is a rare disorder characterized by tumorous overgrowth of the muscular wall of the oesophagus. DOL is present in 5 % of Alport syndrome (AS) patients. AS is a rare hereditary disease that involves varying degrees of hearing impairment, ocular changes and progressive glomerulonephritis leading to renal failure. In DOL-AS patients, the genetic defect consists of a deletion involving the *COL4A5* and *COL4A6* genes on the X chromosome.

**Case presentation:**

We report a two-generation family (4 individuals; parents and two children, one male and one female) with two members (mother and son) affected with oesophageal leiomyomatosis. Signs of potential renal failure, which characterizes AS, were only apparent in the index patient (son) 2 years and three months after the initial diagnosis of DOL. Blood DNA from the four family members were submitted to exome sequencing and array genotyping to perform a genome wide screening for disease causal single nucleotide (SN) and copy number (CN) variations. Analyses revealed a new 40kb deletion encompassing from intron 2 of COL4A5 to intron 1 of COL4A6 at Xq22.3. The breakpoints were also identified. Possible confounding pathogenic exonic variants in genes known to be involved in other extracellular matrices disorders were also shared by the two affected individuals. Meticulous analysis of the maternal DNA revealed a case of gonosomal mosaicism.

**Conclusions:**

This is the first report of gonadosomal mosaicism associated to DOL-AS

**Electronic supplementary material:**

The online version of this article (doi:10.1186/s12881-015-0189-7) contains supplementary material, which is available to authorized users.

## Background

Diffuse oesophageal leiomyomatosis (DOL) is a rare disorder characterized by tumorous overgrowth of the muscular wall of the oesophagus. DOL has been described in 5 % of Alport syndrome (AS) patients reported (DOL-AS) [1, 2]. AS is a rare hereditary disease that involves varying degrees of hearing impairment, ocular changes and progressive glomerulonephritis leading to renal failure. In DOL-AS patients, the genetic defect consists of a large deletion involving the *COL4A5* and *COL4A6* genes on the X chromosome. Thus, the DOL-AS inheritance pattern is that of an X-linked dominant disorder where male patients hemizygous for mutations in *COL4A5* are severely affected, but heterozygous females may display a broad spectrum of symptoms, ranging from none at all to severe renal dysfunction. Fusion of repetitive elements located within introns of *COL4A5* and *COL4A6* may result in long deletions, thus underlying the structural rearrangements observed in DOL-AS patients [3]. The size of the deletion varies among affected individuals although it usually spans from intron 2 of *COL4A6* to intron 1 *COL4A5,* although in some instances, can include the whole *COL4A5* gene [4–8]. The heterogeneity of the deletions observed together with the existence of repetitive elements (LINE-1) in these genes suggests a high occurrence of *de novo COL4A5/COL4A6* deletions [3].

As there are large variations in the phenotype and severity of AS, the disorder may remain undiagnosed. Early diagnosis of AS is essential as current therapy can slow the progression to end-stage renal disease which prompts the need of using molecular tests for more accurate diagnosis. Genetic testing is indeed the gold standard.

In this manuscript we present a two-generation family (4 individuals; parents and two children, one male and one female) with two members (mother and son) affected with oesophageal leiomyomatosis. Signs of potential renal failure, that characterizes AS, were only apparent in the index patient (son) 2 years and three months after the initial diagnosis of DOL.

In order to provide a molecular signature to help diagnose these patients, DNA from all family members was submitted to whole-exome sequencing (WES) for the detection of rare single nucleotide variants (SNVs) and to high density genotyping of common single nucleotide variants polymorphisms (SNPs) for the detection of variation in the number of copies of genomic regions (Copy Number Variants; CNV). This resulted in the identification of a new deletion involving two collagen IV chains, *COL4A5* and *COL4A6* at Xq22.3. Experiments to define the precise breakpoints confirmed the deletion spans from intron 2 of *COL4A5* to intron 1 of *COL4A6*.

### Case presentation

Four members of a non-consanguineous Chinese family were included in the study. The study was approved by the institutional review board of The University of Hong Kong together with the Hospital Authority (IRB: UW 07–321). Blood samples were drawn after obtaining written informed consent. Parents gave written consent on behalf of their two children.

A 13-year-old boy (index patient) who had previously been well was admitted to our medical center because of a history of slowly progressive dysphagia for over 3 years. He also had intermittent episodes of dyspnea within a week of admission. There was no other associated symptom. On further questioning, his mother had also been suffering from mild dysphagia for over 20 years. His father and younger sister were unaffected.

**Fig. 1:**
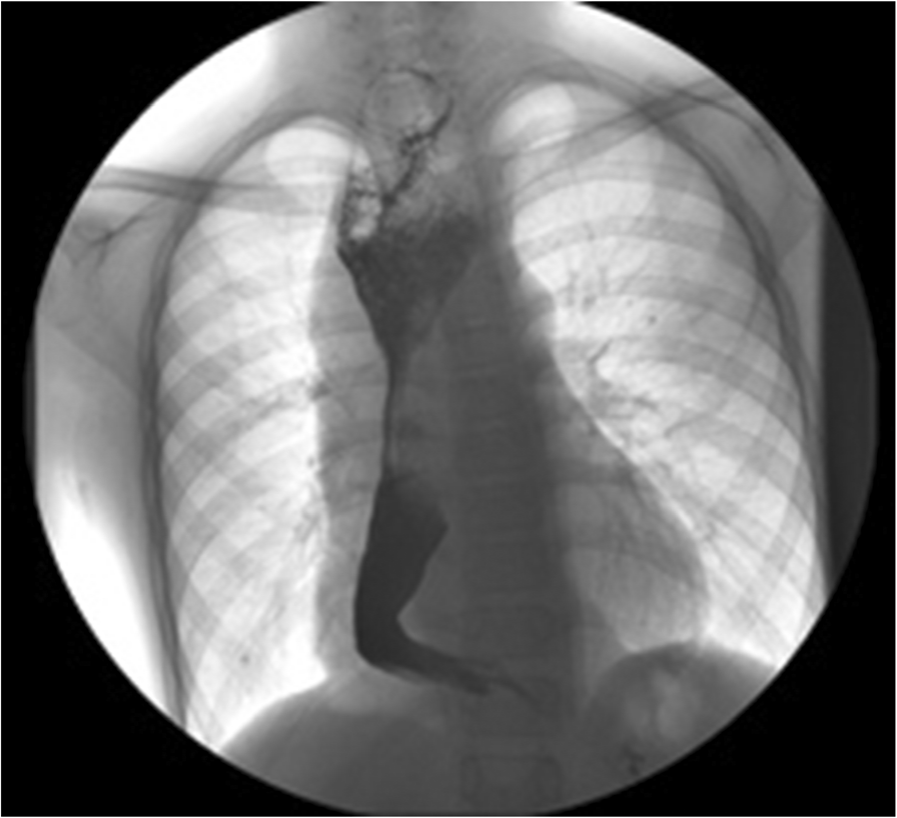
A photo of the contrast esophagogram

**Fig. 2:**
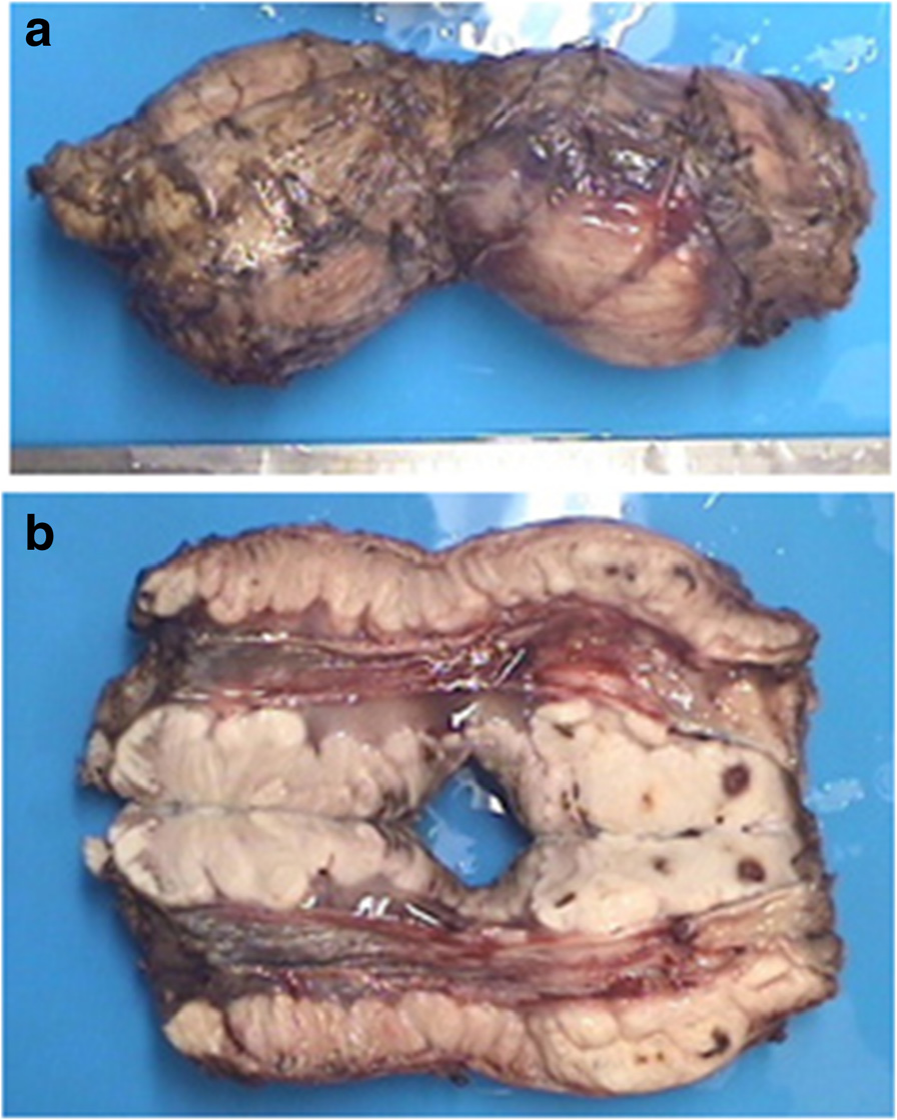
Surgical specimen showing three nodular, well circumscribed nodules (**a**) of solid, dull white tumour with a whorled pattern that circumferentially surrounded the whole esophagus (**b**)

**Fig. 3:**
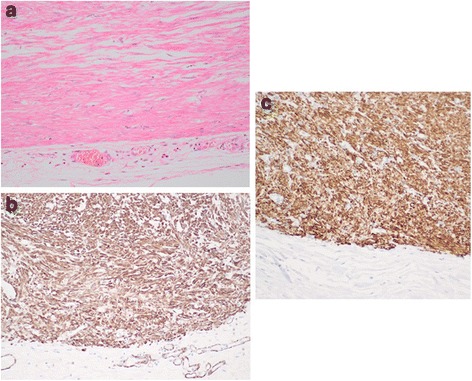
Haematoxylin and eosin section of the tumour showing intersecting fascicles of cytologically benign smooth muscle cells (**a**) that are positive for actin (**b**) and h-caldesom (**c**). Magnification X 200

His body weight was 30 kg and height was 135 cm, both below the average for this age. Physical examination was unremarkable. Blood tests showed mild anemia (Hb 106 g/L, MCV 74.1 fL and MCH 23.3 pg). Chest X-ray showed a widened mediastinal contour. Subsequent contrast esophagogram demonstrated narrowing of middle and distal esophagus (Fig. 1). Contrast-enhanced computed tomography of the chest illustrated a circumferential soft tissue mass encircling the whole esophagus diffusely. Due to the long segment of the esophageal involvement and the possibility of malignant transformation, right-sided thoracotomy was performed. Inspection of the thoracic cavity showed a lotus-root-like esophagus with massive dimension, reaching a maximum diameter of 9 cm. The whole esophagus was resected with reconstruction using a gastric tube intrathoracically. The surgical specimen showed three nodular, well circumscribed nodules of solid, dull white tumour with a whorled pattern that circumferentially surrounded the whole esophagus (Fig. 2A, B respectively). Histology showed interlacing bundles of cytologically benign smooth muscle cells that were positive for smooth muscle actin (Fig. 3). On the basis of these findings, the lesion was diagnosed as leiomyomatosis. There was no evidence of malignancy histologically. His mother also underwent esophagogram and computerised tomography (CT) and the results were very similar to those of her son although X-ray findings do not necessarily correlate with clinical symptoms. Since she had no symptoms, she refused further examination or treatment.

The index patient underwent surgery on January 28th, 2013. On post-operative day one, the patient had hematuria and mild proteinuria caused by urethral catheterization. These completely disappear on postoperative day four. Uric acid/trioxypurine, creatinine clearance and urea were checked to re-assess renal function on postoperative day ten. These parameters were consistent with normal renal function. Neither hearing nor ocular problems were detected in the patient or his mother.

Overall, the patient’s postoperative course was unremarkable and he was discharged 23 days after operation. He remained free of any clinical symptom at 6-month follow-up. However, hematuria and kidney functional abnormalities were detected in the index patient 2 years and three months (April 2015) after the initial DOL diagnosis.

We proceed with molecular testing by scanning all coding sequences of the genome for mutations or CNVs that could account for the phenotype observed in the index patient and his mother.

### Molecular testing

#### Copy number variations

A genome wide screening on the family was achieved by using *Affymetrix* Genome-Wide Human SNP Array 6.0. SNP calls were provided by Center of Genomic Sciences of the University of Hong Kong. SNP Array 6.0 contains 906,600 SNP probes and 946,000 non-polymorphic probes that cover 5,677 known CNV regions in Toronto Database of Genomic Variants. Both types of probes were used for CNV detection and consanguinity analysis. CNVs were called by two programs, *Affymetrix* Genotyping Console 2.1 (GTC) and QuantiSNP which uses a Hidden Markov Mode. For the later, CNV calls were based on normalized probe intensity value against expected intensity in terms of R ratio (LRR) and relative allelic copy in terms of B allele frequency (BAF).

To retrieve high confidence calls, we filtered out non-overlapping CNV regions reported by GTC and QuantiSNP. By intersecting CNV calls from these two calling algorithms, the false positive rate should be minimized.

Since Collagen Type IV is frequently associated with diffuse oesophageal leiomyomatosis, manual screening of CNVs on these genes was performed and normalized probe intensities were visualized by *Affymetrix* Genotyping Console Browser.

#### CNV validation

Copy number validation was performed by quantitative real-time PCR (ABI Prism 7900 Sequence Detection System; Applied Biosystems) using TaqMan® Copy Number Assay. The assay was carried out in quadruplicates with the TaqMan® Copy Number Reference Assay according to the manufacturer’s protocol. The reference assay targets a copy-number neutral region of RNaseP gene, serving as an internal standard. We used Hs01881795_cn which falls on chrX:107,682,713, on exon 1 of the gene *COL4A6*. Relative levels of *COL4A6* to reference probes were determined using comparative C_T_ method by ABI CopyCaller. In brief, the mean differences in cycle threshold (C_T_) ΔC_T_ between the *COL4A6 probe* and the reference probes for all replicates were computed and were subsequently normalized for copy number prediction.

#### Detection of the breakpoint

For the detection of the breakpoint, we designed set of primers spanning ChrX:107685385–107685404 and ChrX:107644439–107644460 (Additional file 1: Table S1). The primer sets were used to amplify the region of interest on the father, affected mother, affected son and the daughter. The PCR amplified product would encompass the deletion junction. Subsequently, PCR products were sequenced to locate the breakpoint.

#### Whole exome sequencing

Exome sequencing was performed in the Centre for Genomic Sciences (CGS) of the Li Ka Shing Faculty of Medicine of the University of Hong Kong. Briefly, Illumina’s TruSeq® DNA Sample Prep v.2 and TruSeq® Exome Enrichment Kits were used for sample preparation and capture (4 libraries per capture reaction) and enrichment of targeted sequences, respectively. The kit captures 62 Mb of the human genome including 20,940 genes, non-coding DNA in flanking regions and regulatory elements. The captured DNA was sequenced as paired-end 100 base reads (PE100) on an Illumina HiSeq 2000, aiming to achieve 121 reads per base (121X) in average (details on variant calling, quality control and data analysis can be found in the Additional file 1).

GATK was used for exome variant calling, exome variant filtration and CNV analysis (see Additional file 1: for details).

#### Pathogenicity criteria for rare variants (SNVs and CNVs)

For SNVs, variant pathogenicity was assessed by an in-house program, KGGSeq. KGGSeq is a software platform constituted of bioinformatics and statistical genetics functions making use of valuable biological resources and knowledge providing a comprehensive and efficient framework to filter and prioritize genetic variants from whole exome and whole genome sequencing data [9, 10]. Importantly, KGGSeq integrates “Knowledge” resources from pseudo-genes and epigenetic databases, biological pathways, OMIM databases and protein-protein interaction networks to annotate the genes that harbour any post-QC variants. KGGSeq also combines deleteriousness prediction (scores and classification) from Polyphen2, Sift, MutationTaster, GERP conservation scores [11] and Likelihood ratio.

Since the oesophagus of the two patients were affected, we also assumed that the causal gene is likely to have high tissue specificity. Therefore annotated the expression of the genes (in which potentially pathological variants had been found) in different tissues. For this, we used RNA-Seq gene expression data from ENCODE and Gene Expression Omnibus (GEO) database. The expression values were used for ranking variants, thus, variants in genes with low expression in esophagus nor involved in developmental processes were down ranked from the list.

Only deleterious nonsynonymous, splice-site and frameshift (SNVs + Indels) variants were further assessed (N = 11,577). Variants with MAF >0.01 in the control populations were filtered out under the assumption that variants underlying this rare disorder would not be frequently found in the general population. A total of 1,013 variants were left for further analysis.

For CNVs, those with >50 % of reciprocal overlap with the population-based Database of Genomic Variants were assessed. CNVs with frequency > 1 % were removed from the analysis.

#### Finding de-novo mutations

KGGSeq also provides a gene filter for searching *de novo* mutations. Since discovery of *de novo* mutations requires accurate detection of genotypes of both parents and the offspring, it is more prone to false positive calls. Thus, we applied a more stringent filter for *de novo* mutations. In addition to the abovementioned filtering criteria applied on rare variants, the following filtering criteria were applied. Each *de novo* mutation requires a read depth > =8X for a callout. Mutations found in any population database were excluded.

## Results

We performed WES and CNV analysis on a two-generation family (4 individuals; parents and two children, one male and one female) with two members (mother and son) affected. While the mother of the index patient was affected isolated DOL, the index patient remained free of the signs that accompany DOL in AS for 2 years and 3 months. Among the genes with variants, we paid particular attention to genes involved in disorders in which leiomyomatosis is part of their phenotypic spectrum (AS).

### Detection of copy number loss on *COL4A5/6*

As DOL may be concomitant with Alport syndrome and all reported DOL-AS cases are associated with mutations in Collagen Type IV genes [5] (in particular with heterogeneous deletions spanning the *COL4A5* and *COL4A6* genes on the X chromosome) [2], we investigated the presence of copy number changes comprising the Collagen Type IV genes by using the SNP Chip data. A deletion comprising exons 1 and 2 of *COL4A5* and exon 1 of *COL4A6* was called by GTC on the son, but only a marginal signal was detected in his affected mother. The deletion spanned from chrX:107,645,023 to 107,685,353 encompassing a total of 40-kb. No such deletion was called in any of the healthy family members.

To further confirm the deletion in the mother, we manually inspected the raw intensity of the SNP chip and employed exome sequencing coverage for detection of copy number changes (Additional file 1: Figures S1 and S2). Variations in the number of copies of exonic sequences can be detected by analyzing and comparing the depth of coverage of the exons in the exome sequencing data. The coverage of the targeted exonic regions were produced by the GATK Depth-of-coverage module. Plotting the exonic coverage along the *COL4A5* and *COL4A6* genes for all family members revealed the aforementioned deletion in the son and again, a marginal detection of such deletion was observed in the maternal exome data.

#### Validation of candidate CNVs

To validate the candidate CNV, a TaqMan® Copy Number assay was performed on the 4 family members. Taqman probes were designed to target exon 1 (on ChrX:107,682,713) of *COL4A6*, which is within the breakpoint detected by SNP Chip. We confirmed a total loss of *COL4A6* exon 1 on the son and a detectable copy number loss on the affected mother (Additional file 1: Figure S3).

#### Breakpoint mapping

**Fig. 4:**
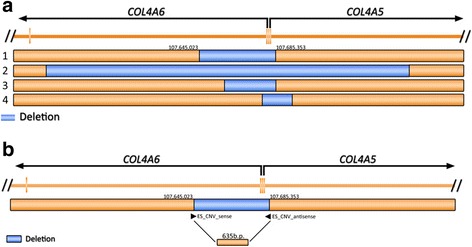
Graphical presentation of previously reported *COL4A5/6* deletions (case 1, current study; case 2 [5] , case 3 [4] , case 4 [3] in –**a**-). The primer set used for accurate detection of the junctions found by Sanger sequencing of the 645b.p. product (**b**)

To fine map the predicted deletion, iterative rounds of PCR were performed with different primer sets. One set (Additional file 1: Table S1) yielded a 635 bp PCR product (instead of the 40,965 predicted) only from the mother and son which indicated that the junction was encompassed there (Additional file 1: Figure S4). Sequencing of PCR products from the mother and the son revealed identical sequence where ChrX: 107,645,023 was next to ChrX:107,685,353 (chrX.hg19:g.107,645,023_107,685,353del) (Fig. 4). The validated CNV has been submitted to the Leiden Open (source) Variation Database with REF numbers #0002653 (https://grenada.lumc.nl/LOVD2/COL4A/variants.php?select_db=COL4A5&action=view&view=0002653) and #0002654 (https://grenada.lumc.nl/LOVD2/COL4A/variants.php?select_db=COL4A5&action=view&view=0002654).

#### Detection of gonosomal mosaicism

As the detection of the deletion was marginal in the maternal blood, we set out to investigate the possibility of gonosomal mosaicism in the mother (Additional file 1). Surprisingly, 3 heterozygous SNPs were detected within the validated deleted region of the affected mother (Additional file 1). These were subsequently confirmed by Sanger sequencing. This observation suggests maternal gonosomal mosaicism for the *COL4A5/6* deletion and explains the results of the maternal blood DNA examination [12]. Indeed, the intermediate level of signal observed across different CNV detection techniques (SNP array, exome sequencing and Taqman CNV assay) indicated that the maternal blood DNA used consisted of cells with two copies of the *COL4A5/6* region and cells with one copy (Additional file 1: Figures. S1-S3).

### Novel SNVs

The data generated by WES had an average coverage (depth) of 90X with over 98 % of the targeted regions exceeding 10X coverage (Additional file 1: Table S2). No contamination signs were detected (Additional file 1: Table S3). Sample relatedness check also indicated normal genetic sharing within the family.

*Under a dominant model of inheritance*, 38 putative deleterious SNVs distributed among 37 genes were selected (Additional file 1: Table S4). None of these SNVs were present in the control populations or public exome databases inspected. Among these 38 SNVs only shared by the affected mother and son, we focused on 3 heterozygous variants mapped to genes (*AGTR1, COL6A3, NOTCH2*) that are associated with congenital disorders whose phenotypic spectrum may include features that could also be present in AS or DOL-AS (Table 1). The three listed mutations were predicted “rare disease causal” by the KGGSeq combined model.

**Table 1:** Top 3 deleterious snv identified by exome sequencing

Chr	Gene	Alleles	Protein	OMIM (#)
3	*AGTR1*	c.502C > T	p.R168*	Renal tubular dysgenesis, #267430
2	*COL6A3*	c.2782C > T	p.R928C	Bethlem myopathy, #158810
				Ullrich congenital muscular dystrophy, #254090
1	*NOTCH2*	c.4758A > C	p.E1586D	Alagille syndrome 2, #610205

*AGTR1* (angiotensin II receptor, type 1) is associated with renal tubular dysgenesis, an autosomal recessive severe kidney disorder characterized by abnormal development of the kidneys before birth. The stop-gain mutation within *AGTR1* (c.502C > T, p.R168*) would terminate the protein at R168 halving the length of the protein which could aggravate the kidney function, already compromised in AS patients [13].

Interestingly, a mutation in a collagen gene (*COL6A3*) (c.2782C > T, p.R928C) was identified. Collagen genes encode structural proteins, collagens, which are the major components of the extracellular matrices (ECMs) of connective tissues. Thus, mutations in *COL* genes result in a wide range of serious inherited disorders (including AS) that although phenotypically diverse, they may share similar disease mechanisms at molecular level. *COL6A3* mutations in particular are associated with congenital muscular disorders such as Ullrich muscular dystrophy and Bethlem myopathy which are characterized by progressive skeletal muscle weakness. The missense mutation identified, Arg928Cys, occurred in a domain region of *COL6A3* that if altered, the stability of collagen monomers formed with alpha-1 and alpha-2 chains could affected [14–16].

*NOTCH2* mutations are associated with Allagile Syndrome and Hajdu-Cheney syndrome, two autosomal dominant conditions. Importantly, a *NOTCH2* mutation has been reported in one case of Allagile Syndrome (autosomal dominant disorder whose main feature is bile duct paucity or even biliary atresia) where the patient also had kidney dysfunction manifestations including hematuria and proteinuria [17]. Likewise, the *NOTCH2* mutation(c.4758A > C, p.E1586D) identified in the index patient and his mother, could be involved in kidney dysfunction although not such sign has been observed.

We also investigated the presence of *de novo* mutations in the index patient as we reasoned that the effect of a maternally inherited mutation could be exacerbated by *de novo* hits and this could account for the differences in the severity of the oesophageal leiomyomatosis between mother and son. Only one *de novo* missense SNVs was found in *C9orf10,* an annotated gene whose function is unknown. This hypothesis was no longer pursued.

## Discussion

We have studied a family with one member (mother of the index patient) affected with isolated diffused esophageal leiomyomatosis (DOL) and another member (index patient), affected with DOL-AS. Importantly, the renal failure signs that characterize AS only appeared 2 years and three months after the initial diagnosis of DOL. By exome sequencing, genotyping array and PCR analysis of patients’ DNA, we have identified a new 40 kb deletion spanning from intron 2 of *COL4A6* to intron 1 of *COL4A5*. Deletions involving *COL4A6/COL4A5* are only found in individuals affected with DOL-AS and no molecular analyses were done on those few individuals with isolated DOL reported in the literature [18–20]. The boundaries of this newly identified deletion are in line with those reported in patients with DOL-AS where the deletion is limited to intron 1 and 2 of *COL4A6*. Deletions whose boundary is beyond exon 3 of *COL4A6* are not seen in DOL-AS syndrome [21]. The affected mother only expressed a mild and isolated DOL and chose not to undergo esophagectomy. In contrast, the son, who carries a hemizygous defective copy of the *COL4A6* gene had a more severe DOL phenotype and esophagectomy was required. X-linked inheritance, together with maternal gonosomal mosaicim may explain the phenotypic differences between mother and son. This would be the first case of gonosomal mosaicism reported in patients (mother) with DOL.

Another interesting fact in this study is the identification, in the affected individuals, of potentially pathogenic mutations in genes known to be involved in other severe congenital disorders (Table 1) including other ECMs disorders. Whether this mutations contribute to the observed phenotype or may exacerbate it later is not known. Noteworthy, a recent study of a five generation family with AS identified a potential confounding variant in the *NPHS1* (nephrin) gene in addition to a *COL4A5* mutation. Likewise, *NPHS1* mutations are associated with nephrotic syndromes and the authors speculate that this mutation could modify the Alport Syndrome phenotype underlain by the *COL4A5* mutation. This may well be the case in the family reported here, yet mutations in the three genes other than *COL4A6* are all maternally inherited. Whether the phenotypic differences between affected mother and son result from the X-link inheritance nature of AS, the gonosomal mosaicism or from the effect of any of those mutations in those genes cannot be assessed [22].

## Conclusions

The family studied is the first case of a gonadosomal mosaicism associated to DOL-AS.

## Additional file

Additional file 1:
**Data analysis and variant calling.**
