# Supplementary material for: Chinese family with diffuse oesophageal leiomyomatosis: a new COL4A5/COL4A6 deletion and a case of gonosomal mosaicism
Source: BMC Med Genet. 2015 Jul 16;16:49. doi: 10.1186/s12881-015-0189-7 (PMC4557859; doi:10.1186/s12881-015-0189-7)
Supplement: Additional file 1: — Data analysis and variant calling. [file 12881_2015_189_MOESM1_ESM.doc]

Contents

[Data analysis and variant calling 2](#__RefHeading___Toc384904161)

[Copy number analysis by whole exome sequencing data 4](#__RefHeading___Toc384904162)

[Heterozygous SNPs detected within the CN Loss region 4](#__RefHeading___Toc384904163)

[**Supplementary Table 1:** Primer set used for breakpoint detection**.** 6](#__RefHeading___Toc384904164)

[**Supplementary Table 2:** Coverage statistics of the four sequenced individuals. 7](#__RefHeading___Toc384904165)

[**Supplementary Table 3**: Cross sample contamination likelihood in terms of FREEMIX values. 8](#__RefHeading___Toc384904166)

[**Supplementary Table 4:** Predicted damaging variants detected by WES 9](#__RefHeading___Toc384904167)

[**Supplementary Table 5:** SNP calls by Affymetrix Human SNP Array 6.0 within the Copy Number Loss region. 11](#__RefHeading___Toc384904168)

[**Supplementary Table 6:** Validation by Sanger sequencing of selected SNPs within the Copy Number Loss region. 12](#__RefHeading___Toc384904169)

[**Supplementary Figure 1:** Coverage plot showing deleted region derived from exome sequencing data. 13](#__RefHeading___Toc384904170)

[**Supplementary Figure 2:** Visualization of normalized CNV probe intensities. 14](#__RefHeading___Toc384904171)

[**Supplementary Figure 3:** Validation of Copy number loss by qPCR. 15](#__RefHeading___Toc384904172)

[**Supplementary Figure 4:** Gel electrophoresis results of the PCR products using primer set (Supplementary table 1). 16](#__RefHeading___Toc384904173)

[**Supplementary Figure 5:** Electropherogram of the two Sanger sequencing validated SNPs. 17](#__RefHeading___Toc384904174)

# Data analysis and variant calling

Calling and filtering (quality control settings specified below) of single nucleotide variants (SNVs) and indels (small insertions/deletions) were performed by the Genome Analysis Toolkit (GATK 2.5-2). Unified-Genotyper module and Variant Quality Score Recalibration (VQSR) module. The calling set contains 4 query samples and 118 samples from patients with familial nasopharynx carcinoma to perform Variant Quality Score Recalibration (VQSR). VQSR compares known sites in variant databases (1000 Genome and dbsnp137) with novel variants in our dataset to investigate relationships between SNPs quality with different sequencing parameters. To find determinants of true SNVs, sequencing parameters such mapping quality, sequencing depth etc were used to discern between true and false positive calls. The effectiveness of these parameters was estimated by the proportion of novel SNVs excluded in all given samples when compared with variant databases (preferably sample size >=30) (see details in supplementary material).

Only the final clean variant sets that pass QA criteria were followed up. These variant sets were also used to examine sample relatedness by calculating IBD/IBS sharing, Mendelian error rate, and homozygosity rate, which are pretty standardized in GWAS and already implemented in PLINK. No quality issues were found in the QA analysis.

Variants were subsequently annotated by KGGSeq, using the reference RefSeq. The 1000 Genome Project (phase 1, v3), the Exome Sequencing Project (ESP6500), the dbSNP_137 were used to assess variant frequency in the control populations. Integrative Genome Viewer (IGV) (www.broadinstitute.org/igv/) was used for visual inspection of the genomic variants.

# Copy number analysis by whole exome sequencing data

As an alternative tool to replicate copy number loss found on the candidate genes (COL4A5 and COL4A6) from Affymetrix Human SNP Array 6.0, reads coverage plots were generated from whole exome sequencing data. Coverage intervals were firstly generated by Depth-of-coverage module of Genome Analysis Toolkit (GATK 2.5-2). The average exonic coverage of the selected regions was extracted from the coverage interval data, and the data was plotted for visual inspection of copy number loss.

# Heterozygous SNPs detected within the CN Loss region

To investigate the possibility of COL4A5/6 deletion mosaicism, genotypes of SNPs within the deleted region were further investigated by the SNP 6 genotyping chip array data. Unexpectedly 3 SNPs within the deleted region were called heterozygous on the mother, and 2 were validated by Sanger sequencing (Supplementary Tables 5 and 6). There are no reported segmental duplication events overlapping with the SNP probes, indicating these SNP probes are unlikely to have multiple targets. The chromatogram produced during Sanger sequencing of the two validated SNPs indicates a difference in signal intensities between full deletion and mosaic (Supplementary Figure 4). We speculate a de-novo COL4A5/6 deletion event on the mother resulted in mosaicism. The exome sequencing coverage, intensities difference in the genotyping chip and rPCR, in comparison of the normal daughter and affected mother also supported the hypothesis.

| Supplementary Table 1: Primer set used for breakpoint detection. | | | | |  |  |
| --- | --- | --- | --- | --- | --- | --- |
| **Primer** | **Sequence** | **Franking region** | **Direction** | **Predicted Size (b.p.)** | | |
| **ES_CNV_sense** | TCACCGATATCT  CCAGCACA | ChrX:108442174  -108442155 | Forward | 40965 | | |
| **ES_CNV_antisense** | TGTGCACATCCT  TTGACATAGT | ChrX:108401209  -108401230 | Reverse |

| Supplementary Table 2: Coverage statistics of the four sequenced individuals. | | | | | | |
| --- | --- | --- | --- | --- | --- | --- |
| **Sample** | **Aligned (%)** | **Average coverage** | **Target >1X** | **Target >4X** | **Target >10X** | **Target >20X** |
| **A** | 99.07% | 94.6 | 99.78% | 99.40% | 98.85% | 97.59% |
| **B** | 99.10% | 92.6 | 99.64% | 99.24% | 98.69% | 97.45% |
| **C** | 99.12% | 93.4 | 99.66% | 99.31% | 98.74% | 97.33% |
| **D** | 99.23% | 82.9 | 99.54% | 99.18% | 98.55% | 97.13% |
| Whole exome sequencing of the family attained high coverage. All family members have >82X mean coverage and >98% targets with over 10X coverage.  *****A: Father, B: Affected mother, C: Affected son, D: Daughter | | | | | | |

| Supplementary Table 3: Cross sample contamination likelihood in terms of FREEMIX values. | |
| --- | --- |
| **Individual** | **FREEMIX value** |
| **A** | 0.00202 |
| **B** | 0.0018 |
| **C** | 0.00162 |
| **D** | 0.00144 |
| The FREEMIX metric indicates percentage of non-reference bases observed on given reference sites. Values smaller than 0.03 in samples indicates cross contaminations unlikely. | |

| Supplementary Table 4: Predicted damaging variants detected by WES | | | | | | |
| --- | --- | --- | --- | --- | --- | --- |
| **Chr** | | **Position** | **Variant** | **Gene** | **Transcript change** | **Type** |
| **1** | | 155291027 | c.253G>T | RUSC1-AS1 | p.A85S | missense |
| **1** | | 196952161 | c.205C>T | CFHR5 | p.R69C | missense |
| **2** | | 210846926 | c.8929G>A | UNC80 | p.V2977M | missense |
| **3** | | 124456758 | c.654A>G | UMPS | p.I218M | missense |
| **3** | | 138763024 | c.439G>T | PRR23C | p.E147* | stopgain |
| **8** | | 55533984 | c.458C>G | RP1 | p.P153R | missense |
| **8** | | 143993482 | c.1426C>G | CYP11B2 | p.L476V | missense |
| **10** | | 128973904 | c.756G>C | FAM196A | p.E252D | missense |
| **11** | | 617520 | c.2351C>T | CDHR5 | p.A784V | missense |
| **11** | | 7950166 | c.44T>C | OR10A6 | p.L15S | missense |
| **14** | | 24027918 | c.297G>C | THTPA | p.R99S | missense |
| **14** | | 95910878 | c.1720G>A | SYNE3 | p.E574K | missense |
| **19** | | 9225868 | c.572A>T | OR7G1 | p.D191V | missense |
| **1** | | 156255720 | c.703C>T | TMEM79 | p.R235C | missense |
| **3** | | 51352444 | c.3287T>C | DOCK3 | p.I1096T | missense |
| **19** | | 35940624 | c.8C>G | FFAR2 | p.P3R | missense |
| **15** | | 42059384 | c.9104A>G | MGA | p.Q3035R | missense |
| **15** | | 72186031 | c.5131G>C | MYO9A | p.G1711R | missense |
| **19** | | 11727662 | c.344C>T | ZNF627 | p.S115L | missense |
| **12** | | 83250836 | c.131C>T | TMTC2 | p.T44M | missense |
| **2** | | 160139262 | c.319C>T | WDSUB1 | p.P107S | missense |
| **1** | | 120466361 | c.4758A>C | NOTCH2 | p.E1586D | missense |
| **20** | | 23065343 | c.1487G>A | CD93 | p.R496H | missense |
| **5** | | 10254290 | c. 139A>G | CCT5 | p.T47A | missense |
| **6** | | 13321235 | c.286C>T | TBC1D7 | p.R96C | missense |
| **19** | | 1487249 | c.746C>T | PCSK4 | p.P249L | missense |
| **3** | | 148459237 | c.502C>T | AGTR1 | p.R168* | stopgain |
| **3** | | 52256416 | c.1916G>A | TLR9 | p.R639H | missense |
| **11** | | 113934554 | c.532G>A | ZBTB16 | p.G178R | missense |
| **1** | | 27101511 | c.4142G>T | ARID1A | p.R1381L | missense |
| **2** | | 238277503 | c.2782C>T | COL6A3 | p.R928C | missense |
| **5** | | 15937289 | c.1470C>G | FBXL7 | p.F490L | missense |
| **10** | | 44874087 | c.264C>A | CXCL12 | p.N88K | missense |
| **11** | | 6652619 | c.3695C>T | DCHS1 | p.P1232L | missense |
| **6** | | 44145024 | c.1283G>T | CAPN11 | p.G428V | missense |
| **13** | | 113818925 | c.538C>A | PROZ | p.L180I | missense |
| **20** | | 22563536 | c.326G>A | FOXA2 | p.S109N | missense |
| **11** | | 1651199 | c.130_131ins  GGCTGT  GGCTCC | KRTAP5-5 | p.G44_45  insGCGS | nonframeshift |
|  | Under the hypothesis of dominant variants shared by the two affected individuals within the family, 38 SNVs passed the genotype filter. All variants were predicted rare disease causal by KGGSeq combined rare disease model. | | | | | |

| Supplementary Table 5: SNP calls by Affymetrix Human SNP Array 6.0 within the Copy Number Loss region. | | | | | | |
| --- | --- | --- | --- | --- | --- | --- |
| **Chr** | **Position** | **Father Genotype** | **Mother Genotype** | **Son**  **Genotype** | **Daughter genotype** | **RSID** |
| **ChrX** | 107655659 | BB | NoCall | / | NoCall | rs714707 |
| **ChrX** | 107656192 | NoCall | AB | / | NoCall | rs5929123 |
| **ChrX** | 107660865 | AA | AB | / | AA | rs5973876 |
| **ChrX** | 107671022 | BB | BB | / | BB | rs7060905 |
| **ChrX** | 107678345 | BB | AB | / | AB | rs7882619 |
| **ChrX** | 107684507 | BB | BB | / | BB | rs16985479 |
| To investigate the possibility of mosacism, SNPs within the validated deletion was called from the SNP chip. Presence of heterozygous SNPs within the deletion supported the hypothesis. | | | | | | |

| Supplementary Table 6: Validation by Sanger sequencing of selected SNPs within the Copy Number Loss region. | | | | |
| --- | --- | --- | --- | --- |
|  | **A (Father)** | **B (Mother)** | **C (Patient)** | **D**  **(sister)** |
| **rs5929123** | AA | AG | / | AG |
| **rs5973876** | GG | GG | / | GG |
| **rs7882619** | GG | GT | / | GT |
| Heterozygous SNPs reported in Supplementary Table 5 were validated by Sanger sequencing, 2 out of 3 SNPs were successfully validated. | | | | |

# Supplementary Figure 1: Coverage plot showing deleted region derived from exome sequencing data.

This coverage plot was generated by GATK Depth of coverage module, average per base coverage were calculated for each exon.

Total deletion on the son was detected where the mother only has marginal decrease in coverage (Black arrows). (A) Father, (B) Affected mother, (C) Affected son, (D) Daughter


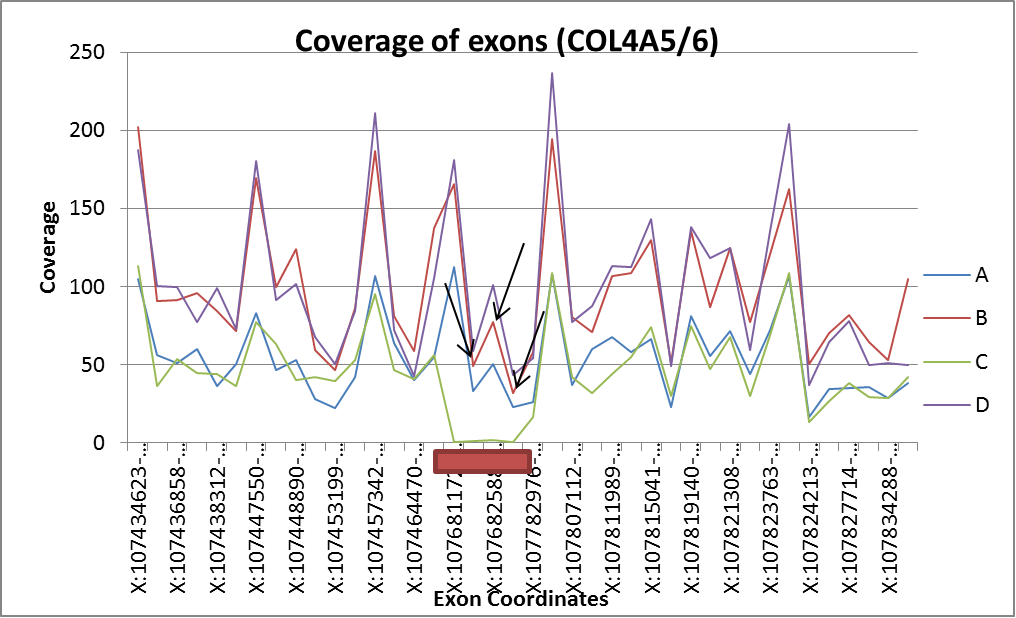


# Supplementary Figure 2: Visualization of normalized CNV probe intensities.

Normalized probe intensities of the SNP array were visualized using Affymetrix Genotyping Console. The deletion was detected on the son. Only marginal signal was detected on the mother (underlined in red).


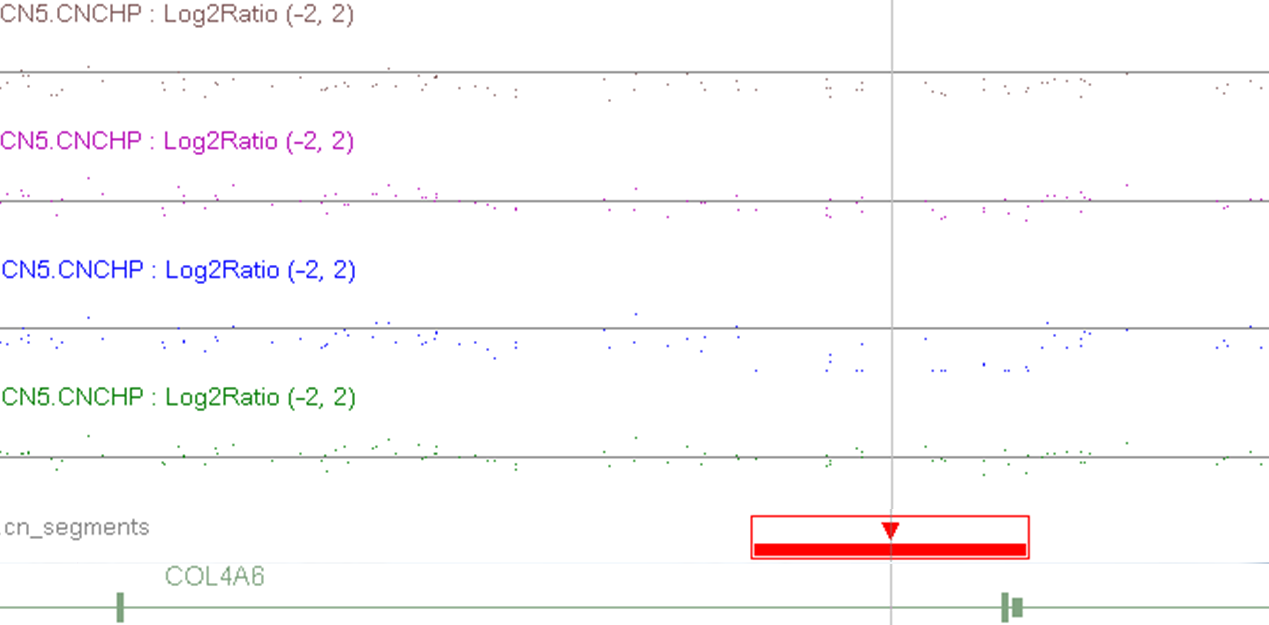


A: Father

B: Affected Mother

C: Affected SON

D: Daughter

# Supplementary Figure 3: Validation of Copy number loss by qPCR.

qPCR was performed on a probe flanking exon 1 of *COL4A6*. Complete deletion was found on the son while ambiguous signal was detected on the mother.


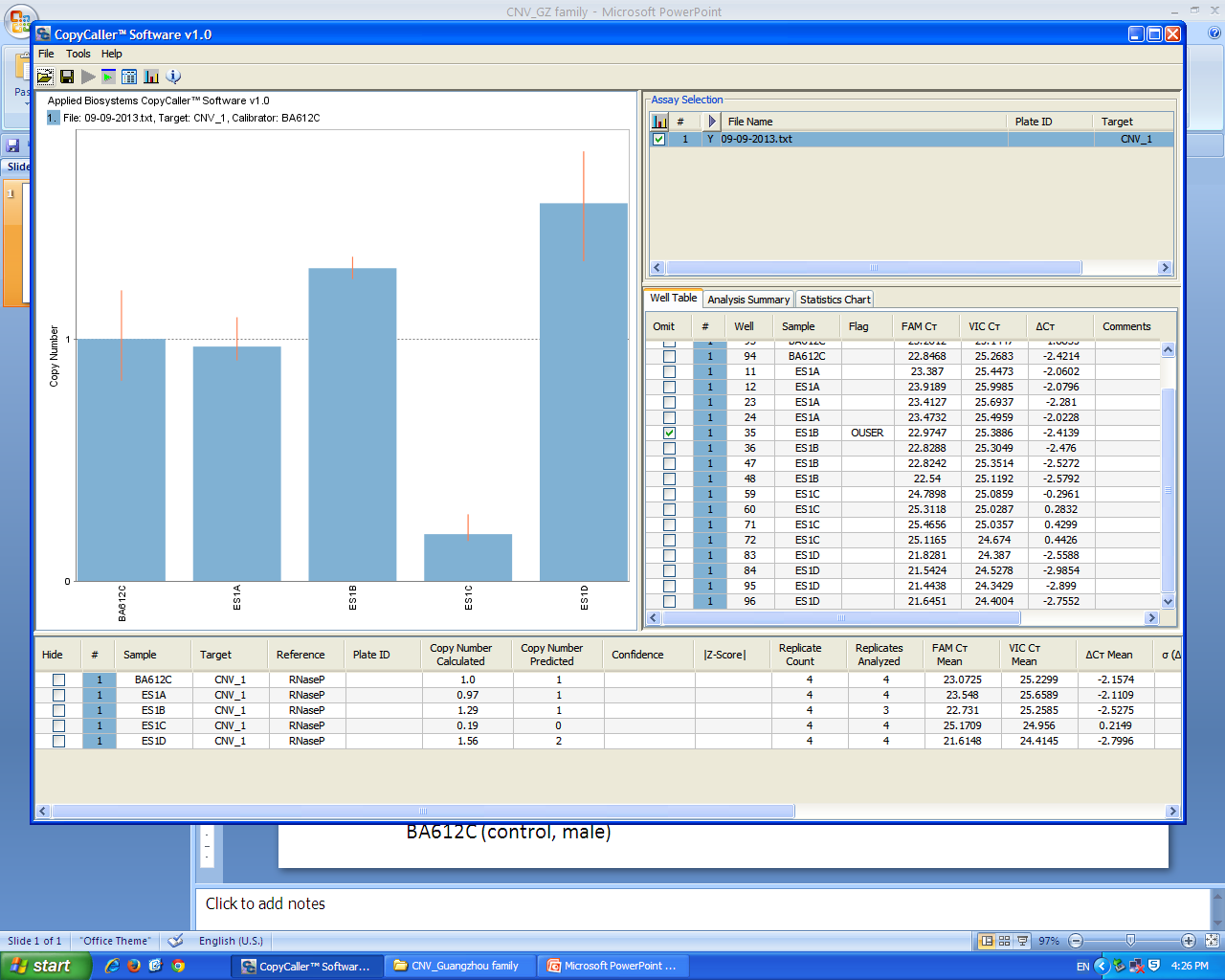


Control

Father

Mother

Son

Daughter

1

1

1/2

0

2

# Supplementary Figure 4: Gel electrophoresis results of the PCR products using primer set (Supplementary table 1).

PCR products were only detected in the affected mother and the affected son. No amplification indicates that the size of the PCR product is too large for the amplification process. Moreover, product size of mother and son is identical, indicated the deletion only present in the two affected members of the family.


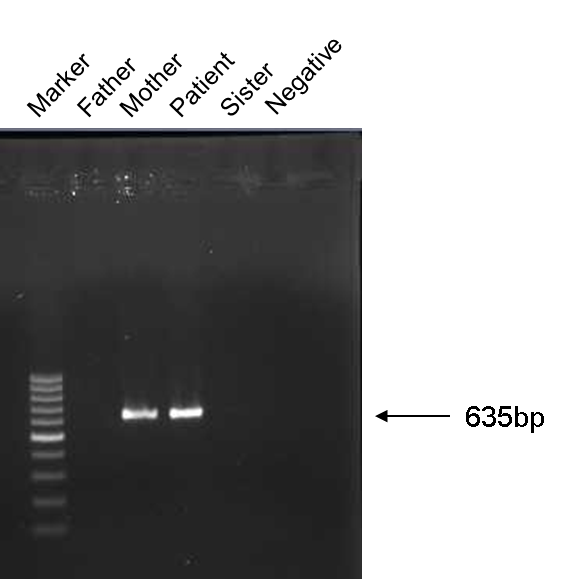


#

# Supplementary Figure 5: Electropherogram of the two Sanger sequencing validated SNPs.

Electropherogram of the two validated heterozygous SNPs within the deleted region has different intensities ratio compared with the normal daughter, showing hints of mosaicism.


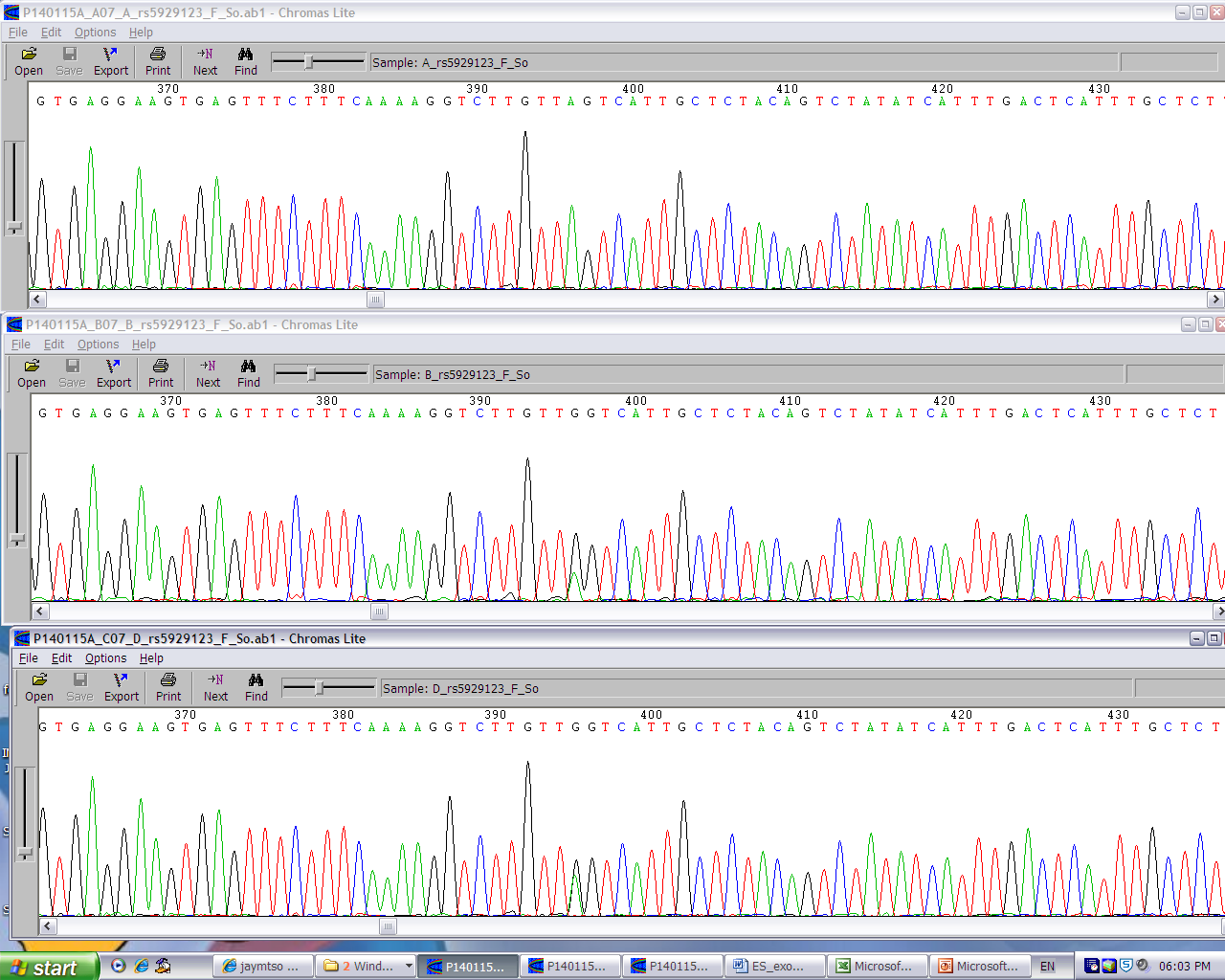


Father

Mother

Sister

**rs5929123**


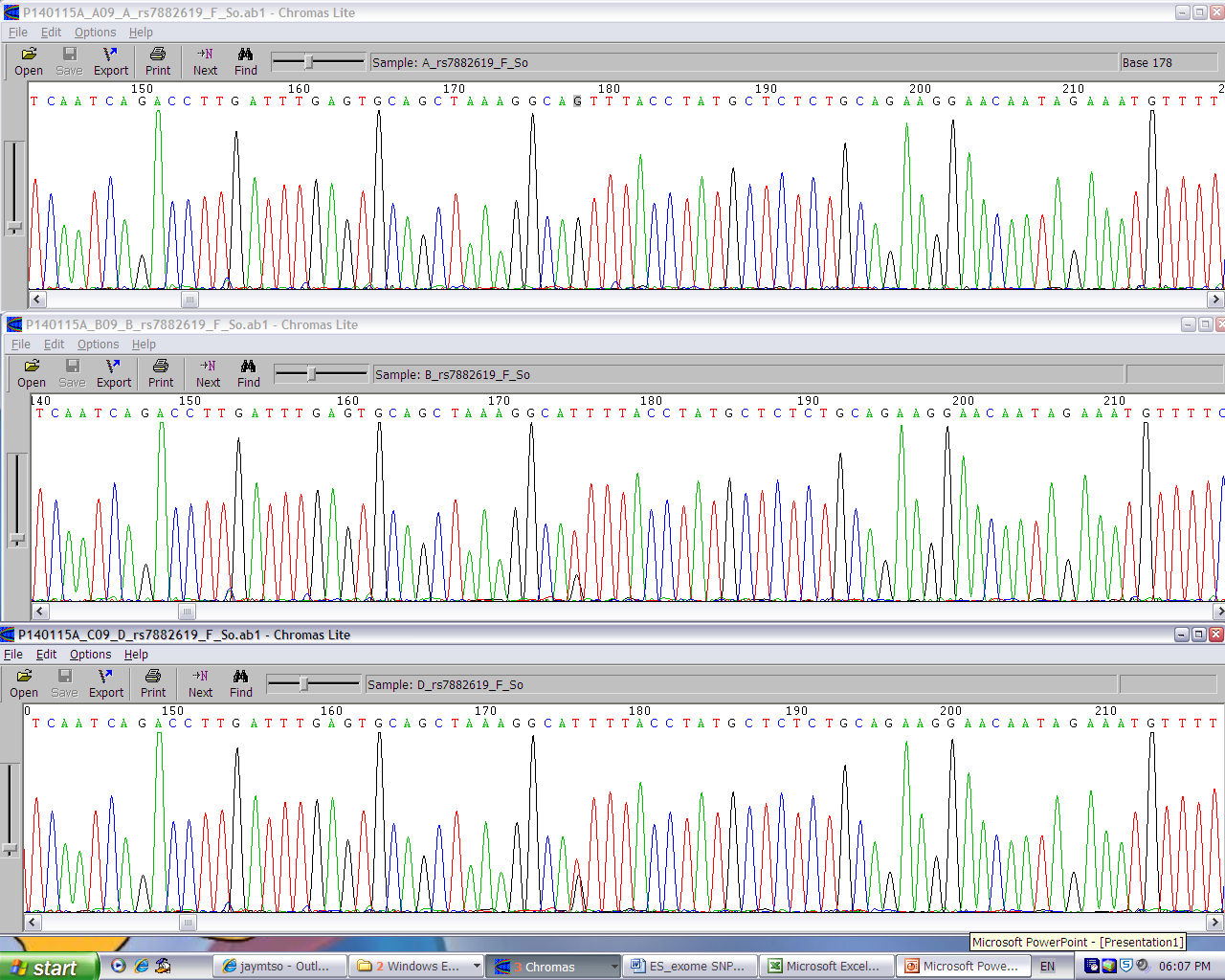


Father

Mother

Sister

**rs7882619**

Father

Mother

Sister

Father

Mother

Sister
